# Anti-Diabetic Effects of Allulose in Diet-Induced Obese Mice via Regulation of mRNA Expression and Alteration of the Microbiome Composition

**DOI:** 10.3390/nu12072113

**Published:** 2020-07-16

**Authors:** Youngji Han, Eun-Young Kwon, Myung-Sook Choi

**Affiliations:** 1 Department of Food Science and Nutrition, Kyungpook National University, 1370 San-Kyuk Dong Puk-Ku, Daegu 702-701, Korea; youngji.kor.han@gmail.com (Y.H.); eykwon@knu.ac.kr (E.-Y.K.); 2 Center for Food and Nutritional Genomics Research, Kyungpook National University, 1370 San-Kyuk Dong Puk-Ku, Daegu 702-701, Korea

**Keywords:** anti-diabetes, anti-obesity, functional sweetener, rare sugar

## Abstract

Allulose has been reported to serve as an anti-obesity and anti-diabetic food component; however, its molecular mechanism is not yet completely understood. This study aims to elucidate the mechanisms of action for allulose in obesity-induced type 2 diabetes mellitus (T2DM), by analyzing the transcriptional and microbial populations of diet-induced obese mice. Thirty-six C57BL/6J mice were divided into four groups, fed with a normal diet (ND), a high-fat diet (HFD), a HFD supplemented with 5% erythritol, or a HFD supplemented with 5% allulose for 16 weeks, in a pair-fed manner. The allulose supplement reduced obesity and comorbidities, including inflammation and hepatic steatosis, and changed the microbial community in HFD-induced obese mice. Allulose attenuated obesity-mediated inflammation, by downregulating mRNA levels of inflammatory response components in the liver, leads to decreased plasma pro-inflammatory marker levels. Allulose suppressed glucose and lipid metabolism-regulating enzyme activities, ameliorating hepatic steatosis and improving dyslipidemia. Allulose improved fasting blood glucose (FBG), plasma glucose, homeostatic model assessment of insulin resistance (HOMA-IR), and the area under the curve (AUC) for the intraperitoneal glucose tolerance test (IPGTT), as well as hepatic lipid levels. Our findings suggested that allulose reduced HFD-induced obesity and improved T2DM by altering mRNA expression and the microbiome community.

## 1. Introduction

Recent and rapid advancements have contributed to increases in the human lifespan. However, a significant proportion of adults suffer from metabolic diseases, caused by fundamental problems associated with diet and lifestyle, making healthcare and treatment a social issue [1]. The worldwide prevalence of obesity has progressively increased over the past 30 years. The World Health Organization estimates that approximately 13% of the world’s adult population was obese in 2016 [2]. The surplus energy intake causes overweight and obesity, inducing increased blood glucose concentration and dyslipidemia, which are obesity complications [3].

Type 2 diabetes mellitus (T2DM) is a metabolic disorder that is primarily caused by obesity-induced insulin resistance, and more than 90% of individuals with T2DM are overweight or obese [4]. Gut microbiota has also captured attention in the last decade, as an element that can directly affect the health or disease status of an individual [5]. Obesity and T2DM have also been related with specific changes in gut microbiota composition [6,7]. Overall, T2DM is associated with elevated levels of pro-inflammatory cytokines, chemokines and inflammatory proteins [8,9]. While some gut microbes and microbial products, especially lipopolysaccharides (LPS), promote metabolic endotoxemia and low-grade inflammation, others stimulate anti-inflammatory cytokines and chemokines [10,11,12,13]. For example, many studies have found a reduced abundance of butyrate-producing species in T2DM patients, leading to a low-grade inflammation in the gut [14], which has been reported in a variety of races and ethnicities and persists after controlling for the effects of anti-diabetic drugs on the gut microbiome [15].

Previous studies have indicated that sugar is a dietary factor that is responsible for obesity and T2DM in modern society [16,17,18]. The huge consumption of carbohydrates finally leads to their storage as triglycerides in the body [19]. Sugar substitutes have received increasing attention [18,20,21], and many studies have provided evidence of the impacts of allulose, a sugar substitute, on diet-induced obesity and its complications [22,23,24,25,26]; however, the anti-diabetic mechanism underlying the transcriptional and microbial actions of allulose remain unclear. Thus, we instigated an animal experiment and analyzed both the changes in transcript expression levels and the biochemical composition of the microbiome, based on the differential abundance in genera.

## 2. Materials and Methods

### 2.1. Animals and Diet

Four-week-old male C57BL/6J mice (Jackson Laboratory, Bar Harbor, ME, USA) were housed in a controlled environment (20–23 °C, alternating 12 h light/dark periods) with free access to water. The mice were fed a commercial diet for one week after arrival for acclimation, then randomly divided into four groups (*n* = 9) and fed the respective experimental diets for 16 weeks, as shown in Table S1. The mice fed a normal diet (ND, 5% fat, w/w), high-fat diet (HFD, 20% fat and 1% cholesterol, w/w), 5% erythritol (ERY, sucrose in HFD substituted with 5% erythritol, w/w), or 5% allulose (ALL, sucrose in HFD substituted with 5% D-allulose, w/w) in a pair-fed condition based on the ALL group and during the experimental period. The procedures of this study were validated by the Ethics Committee for Animal Studies at Kyungpook National University, Daegu, Republic of Korea (Approval No. KNU-2016-130).

### 2.2. Plasma Biochemical Profile

Plasma triglycerides (TG), total cholesterol (total-C) non high-density lipoprotein-cholesterol (HDL-C) were measured using an Asan enzymatic kit (Asan, Seoul, South Korea). Plasma free fatty acid (FFA), apolipoprotein A1 (ApoA1), and apolipoprotein B (ApoB) levels were determined using a commercially available enzymatic kit (Nittobo Medical Co., Tokyo, Japan). The value of nonHDL-cholesterol (nonHDL-C) was calculated as follow: nonHDL-C = (TC)-(HDL-C).

We used a multiplex detection kit from Bio-Rad (Hercules, CA, USA) for measuring plasma insulin, glucagon-like peptide 1 (GLP-1), gastric-inhibitory polypeptide (GIP), glucagon, and adipokine (leptin, resistin, and adiponectin). All samples were assayed in duplicate and data analyses were performed using Bio-Plex Manager software, version 4.1.1 (Bio-Rad, Hercules, CA, USA).

### 2.3. Fasting Blood Glucose, Intraperitoneal Glucose Tolerance Test, and Homeostatic Index of Insulin Resistance

Animals were fasted for 12 hours prior to undergoing a fasting blood glucose determination test (FBG) and an intraperitoneal glucose tolerance test (IPGTT). FBG concentration was measured using a OneTouch Select Plus^®^ meter glucose analyzer (LifeScan, Milpitas, California) from the tail veins. IPGTT was performed at the 11th week. Glucose was intraperitoneally injected at 0.5 g·per·kg of body weight and blood glucose concentrations were determined at 0, 30, 60 and 120 min. The homeostatic index of insulin resistance (HOMA-IR) was calculated according to the following formula: HOMA-IR = (fasting glucose (mmol/L) × fasting insulin (µL·U/mL))/22.5 [27].

### 2.4. mRNA-Seq Experiment

HiSeq Illumina sequencing was performed commercially (LAS, Gimpo, South Korea) using hepatic tissue. RNA-seq libraries were prepared from total RNA using the TruSeq Stranded mRNA Sample Preparation Kit. The library was sequenced on an Illumina Nextseq 500 sequencer using paired-end run (2 × 75 bases) and its detailed protocol was provided by Illumina (https://sapac.illumina.com/).

### 2.5. Histopathology Analysis

The liver and pancreas were collected and immediately fixed using a buffer solution of 10% (v/v) formalin. Liver and pancreas sections were cut at 4 μm-thick. The hematoxylin and eosin (H&E) and Masson’s trichrome (MT) stain were performed. All stained areas were viewed using an optical microscope (Nikon, Tokyo, Japan) with a magnifying power of 200×.

### 2.6. Microbial Community Analysis

Genomic DNA was extracted from mouse feces using the QIAamp DNA Stool Mini Kit (Qiagen) and was analyzed with the Illumina Miseq System. Qiime’s pipeline for preprocessing was used [21]. The demultiplexing and quality filter was employed in the first step (Phread ≥ Q20). Then, using the split reads, chimera detection was applied through usearch (https://www.drive5.com/ usearch/) and the green genes database (https://greengenes.secondgenome.com/) [28,29].

### 2.7. Statistical Analysis

Phenotype and microbiome data were expressed as the mean ± standard error (SE) or standard deviation (SD). Statistical differences between the ND and HFD results were performed by the Mann Whiteny U t-test. A Kruskal-Wallis test was determined among the HFD groups, and Dunn’s Multiple Comparison test was followed post hoc. Data with different superscript letters are significantly different according to the post hoc Kruskal-Wallis test (*p* < 0.05). We used the SPSS software for analysis (SPSS, Inc., Chicago, IL, USA). mRNA expression profiling analysis employed two ANODEV models: (1) two-way ANODEV and (2) simple ANODEV, to analyze and filter out the optimal candidate differentially expressed genes (DEGs) with multiple testing corrections using false-discovery rate (FDR) [29,30].

## 3. Results

To investigate the effects of long-term allulose supplementation in diet-induced obese mice, five-week-old male C57BL/6J mice were provided with HFD with or without sugar substitute supplements for 16 weeks. ALL group was compared with sugar and erythritol supplemented groups. As stated in the method section, all diets except ND group were isocaloric diets that were pair fed based on food intake of ALL group.

### 3.1. Allulose Lowered Body Fat and Plasma Lipid Levels in Diet-Induced Obese (DIO) Mice

**Figure 1:**
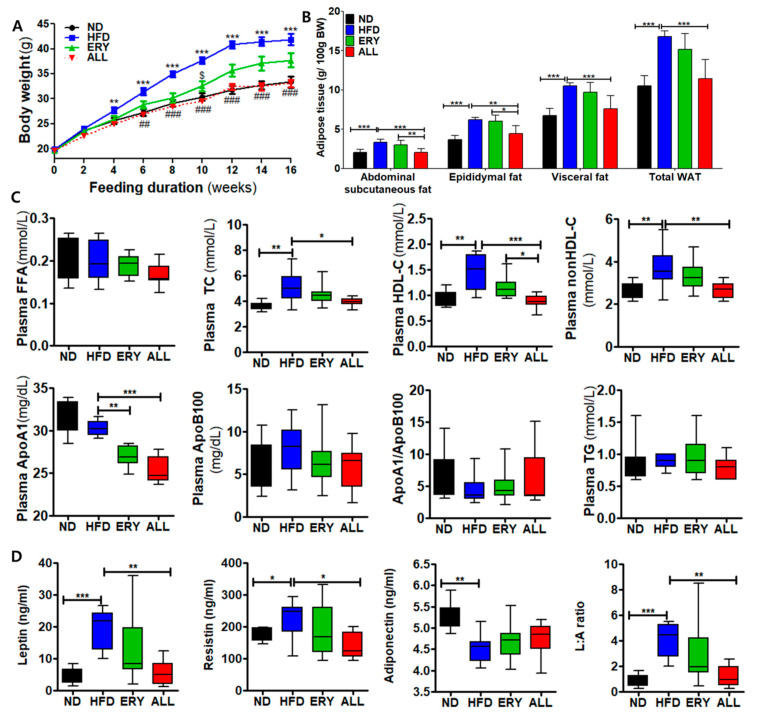
Effects of D-allulose supplement for 16 weeks, on body and adipocyte weights and plasma lipid levels, in C57BL/6J mice fed a high-fat diet. (**A**) Body weight. (**B**) Adipocyte weights. (**C**) Plasma lipid levels. (**D**) Adipokine levels. Data are presented as the mean ± SE. Kruskal-Wallis test; * *p* < 0.05, ** *p* < 0.01, *** *p* < 0.001 versus control; ND, normal diet; HFD, high-fat diet (20% fat, 1% cholesterol); ERY (HFD + 5% Erythritol); ALL, (HFD + 5% D-allulose); FFA, free fatty acid; TC, total cholesterol; HDL-C, high-density lipoprotein cholesterol, ApoA1, apolipoprotein A1; ApoB, apolipoprotein B; non-HDL-C = (TC) – (HDL-C), L:A ratio, leptin: adiponectin ratio.

Changes in body weight over the 16-week experimental period are shown in Figure 1A and Table 1. The initial mouse body weights were not significantly different among the four groups. During the 16-week experimental diet period, ALL group mice showed a significant decrease in body weight compared with the HFD group, starting in week 6 and with similar values to those observed for the ND group. The food efficiency ratio was significantly decreased in the ALL group compared with the HFD group, although no significant difference in food intake was observed between the HFD group and the ALL group. The ERY group demonstrated significantly decreased food and energy intake compared with the HFD group.

After being sacrificed, adipose tissue was collected from mice and weighted (post-mortem determination). Adipose tissue weight was applied per 100 g body weight (Figure 1B). All adipose tissue weights were significantly increased by HFD feeding. However, the ALL group showed significant reductions in abdominal subcutaneous, epididymal, and visceral fat weights and total white adipose tissue (WAT) compared with the HFD group.

Changes in plasma lipid profiles are indicated in Figure 1C. No significant differences in plasma FFA, TG, Apo B, or the Apo A1/Apo B ratio were observed among all groups. HFD feeding induced significant increases in plasma Total-C, HDL-C, non-HDL-C, and Apo A1 levels compared with those in the ND group; however, the ALL group showed lower values for these variables than those in the HFD group.

### 3.2. Allulose Lowered Insulin Resistance and Glucose Tolerance by Modulating the Activities of Hepatic Glucose-Regulating Enzymes in DIO Mice

**Figure 2:**
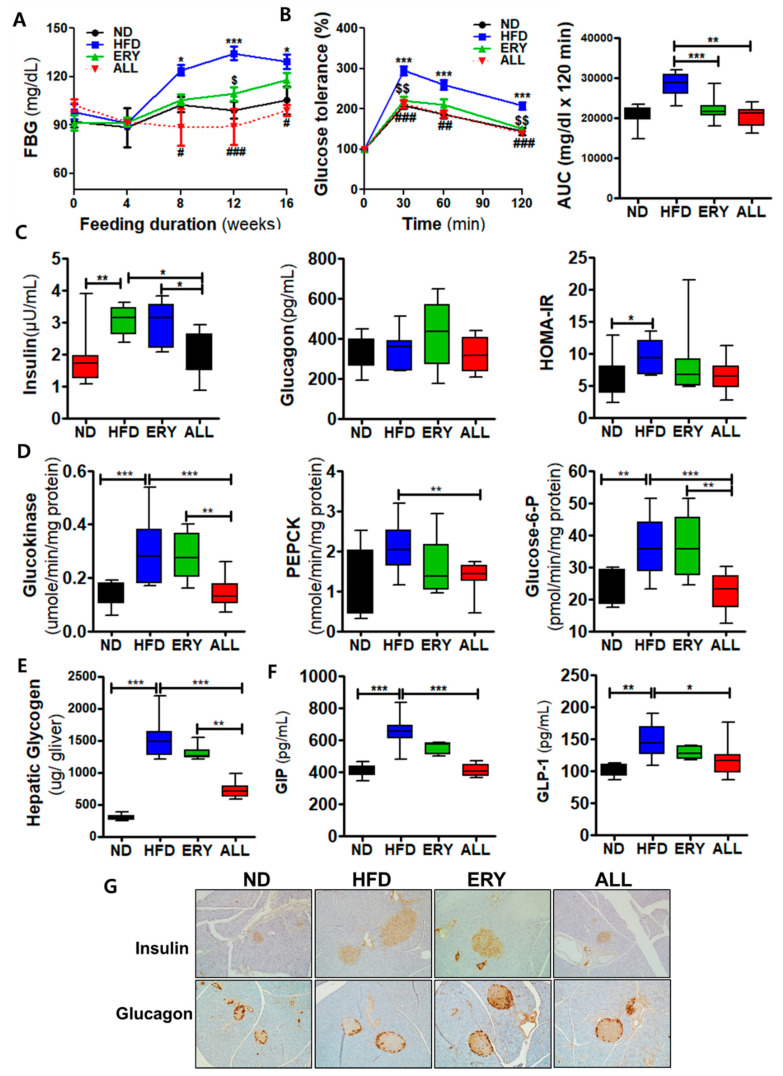
Effect of 16-week D-allulose supplementation on insulin resistance-related biomarkers in C57BL/6J mice fed a high-fat diet. (**A**) Changes in FBG levels. (**B**) Intraperitoneal glucose tolerance test and area under the curve (AUC). (**C**) Plasma insulin, glucagon, and homeostatic model assessment of insulin resistance (HOMA-IR). (**D**) Hepatic glucose metabolism-related enzyme activity. (**E**) Plasma gastric-inhibitory polypeptide (GIP) and glucagon-like peptide 1 (GLP-1) levels. (**F**) Hepatic glycogen. (**G**) Immunohistochemistry staining for insulin and glucagon in the pancreas (200× magnification); Data are presented as the mean ± SE. Kruskal-Wallis test; * *p* < 0.05, ** *p* < 0.01, *** *p* < 0.001 versus control; ND, normal diet; HFD, high-fat diet (20% fat, 1% cholesterol); ERY (HFD + 5% Erythritol); ALL, (HFD + 5% D-allulose); FFA, free fatty acid; TC, total cholesterol; HDL-C, high-density lipoprotein cholesterol, ApoA1, apolipoprotein A1; ApoB, apolipoprotein B; non-HDL-C = (TC) – (HDL-C).

The FBG and plasma insulin concentrations were markedly decreased in the ALL group compared to HFD group starting in week 4 (Figure 2A). The IPGTT and AUC results showed that significantly improved glucose tolerance in the ALL group compared to HFD group (Figure 2B) Furthermore, plasma insulin levels were significantly decreased in the ALL group compared with the HFD group (Figure 2C). Hepatic phosphoenolpyruvate carboxy-kinase (PEPCK), glucokinase (GK), and glucose-6-phosphate (G6pase) activities were significantly decreased in the ALL group, and hepatic glycogen levels were also lowered in the ALL group compared to HFD and ERY group (Figure 2D). Plasma GLP-1 levels in ALL group were lower than HFD group, while plasma GIP levels in ALL group were higher than HFD group. (Figure 2F). The immunohistochemistry stain analyses of pancreatic insulin and glucagon described are shown in Figure 2G. ALL supplement ameliorated the hypertrophy of islets cells induced by DIO, whereby we see the decrease in pancreatic insulin and glucagon content, consistent with the plasma insulin and glucagon concentration results (Figure 2B).

### 3.3. Allulose Reduced Hepatic Lipid Accumulation and Fibrosis by Modulating the Inflammatory Response in DIO Mice

**Figure 3:**
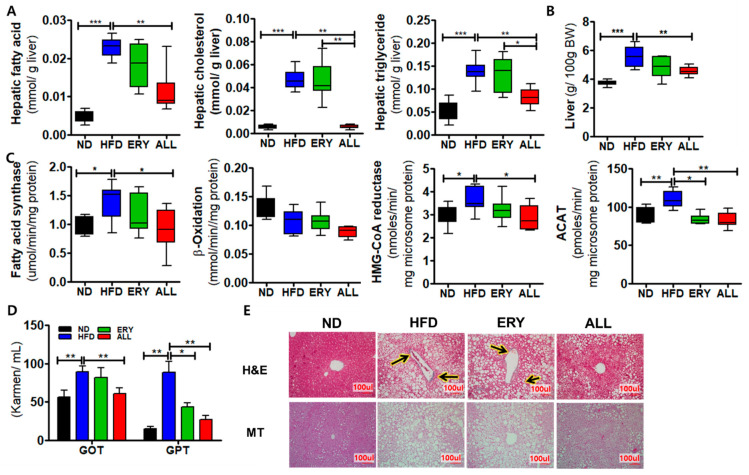
Effects of 16-week D-allulose supplement on hepatic steatosis-related markers in C57BL/6J mice fed a high-fat diet. (**A**) Hepatic lipid contents. (**B**) Liver weights. (**C**) Hepatic lipid metabolism-related enzyme activities. (**D**) Masson’s trichrome staining; (**E**) Plasma GOT and GPT levels; Data are presented as the mean ± SE. Kruskal-Wallis test; * *p* < 0.05, ** *p* < 0.01, *** *p* < 0.001 versus control; ND, normal diet; HFD, high-fat diet (20% fat, 1% cholesterol); ERY (HFD + 5% Erythritol); ALL, (HFD + 5% D-allulose); HMG-CoA reductase, hydroxy-methyl-glutaryl-coenzyme A reductase; ACAT, Acyl-CoA: cholesterol acyltransferase; GOT, glutamic-oxaloacetic transaminase; GPT, glutamic-pyruvic transaminase. Arrow indicates fibrosis signs (blue).

The ALL group showed significantly decreased hepatic lipid contents and liver weights, which were increased in the HFD group (Figure 3A,B). The ALL group also showed significantly decreased lipid metabolism-related enzymatic activities, including fatty acid synthase (FAS), β-oxidation, β-hydroxy-β-methyl-glutaryl-coenzyme A (HMG-CoA) reductase, and acyl-CoA: cholesterol acyltransferase-1 (ACAT) Figure 3C. In accordance with these results, the morphological observations of lipid formation in the liver tissue showed reduced lipid droplet formation in the liver of the ALL group (Figure 3E).

**Figure 4:**
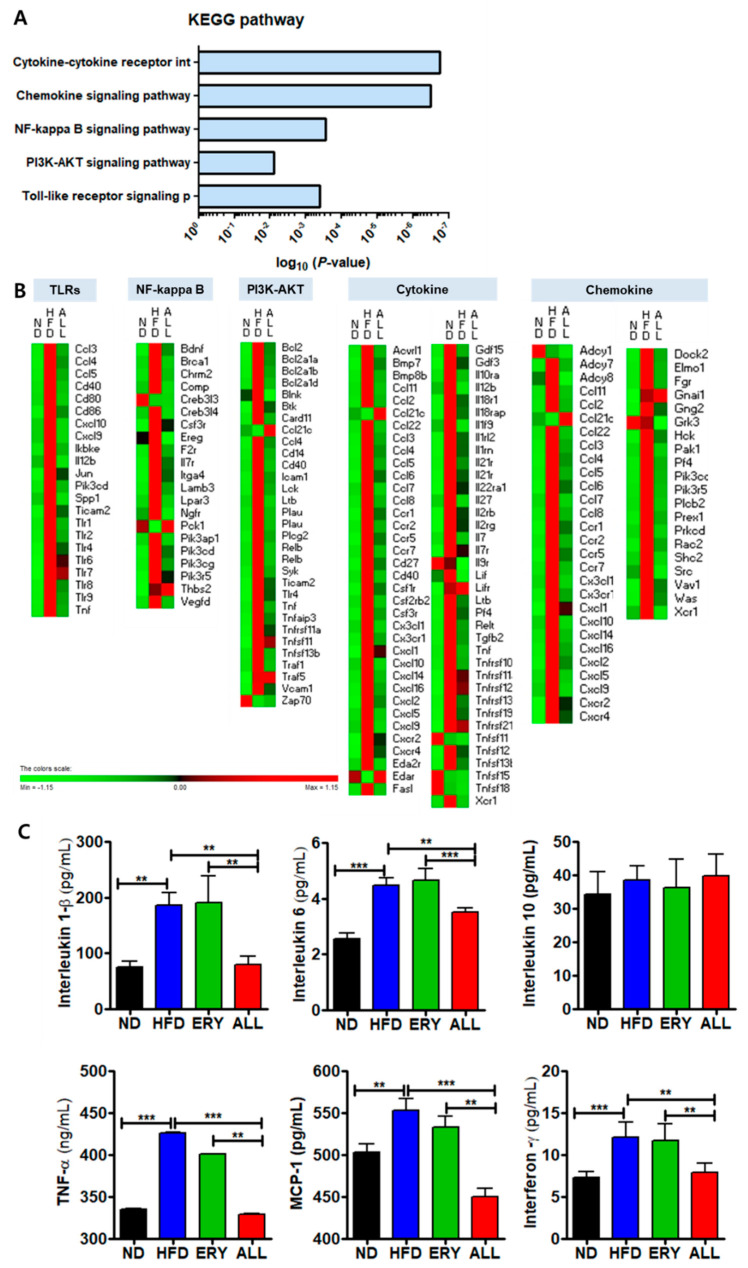
Gene transcription patterns related to inflammation in hepatic tissue. (**A**) Significant KEGG pathways. (**B**) The heatmap shows genes associated with the Toll-like receptor, NF-kappaB, PI3K-AKT, cytokine, and chemokine pathways. (**C**) Plasma inflammatory cytokine levels. Symbols in red were upregulated, whereas those in green were downregulated. Significant differences among the experiment groups are indicated; * *p* < 0.05, ** *p* < 0.01, *** *p* < 0.001; ND, normal diet (AIN-76); HFD, high-fat diet (AIN-76, 20% fat, 1% cholesterol); ERY (HFD + 5% Erythritol); and ALL, (HFD + 5% D-allulose).

The ALL group showed significantly reduced plasma leptin and resistin levels and a reduced leptin: adiponectin (L:A) ratio, whereas the plasma adiponectin level was significantly increased. Moreover, the inflammatory cytokine concentrations, containing interleukin (IL)-1β, IL-6, interferon (IFN)-γ, monocyte chemoattractant protein 1 (MCP1), and tumor necrosis factor (TNF)-α, were markedly decreased in the ALL group compared with those in the HFD group (Figure 4C). As shown in Figure 3E, we performed MT staining to investigate fibrosis and collagen accumulation in the liver. The MT staining showed the increased greater accumulation of collagen in the livers of the HFD group compared with those in the ND group. However, the ALL group showed reduced accumulation of collagen compared with the HFD group and a similar state to that of the ND group. The levels of plasma GOT and GPT, hepatic lipo-toxicity markers, were significantly decreased in the ALL group compared with the HFD groups (Figure 3D).

### 3.4. Allulose Altered the Transcriptional Responses in the Liver Tissue of DIO Mice

RNA sequencing (mRNA-seq) analysis was performed to investigate the transcriptomic profile of the liver tissue. Differentially expressed genes (DEGs) were identified using the following cut-off: fold change (FC) ≥ 2, false-discovery rate (FDR) < 0.05 (Table S2).

In the liver, HFD supplement up-regulated 1285 DEGs and down-regulated 370 DEGs compared with the ND group. HFD with ALL supplement up-regulated 201 genes and down-regulated 292 genes compared with the HFD group. In addition, ALL group up-regulated 178 DEGs and down-regulated 812 DEGs relative to the ND group. Additional details regarding the DEGs can be found in Table S3.

There was no significant difference in food efficiency ratio (FER) between HFD group and ERY group, although body weight in ERY group decreased compared with HFD group. These results suggested that the weight loss effect in ERY group is due to the decrease in food intake. That is the reason why we have not performed the mRNA-seq on the ERY group.

To analyze the transcriptional pattern and biological pathways associated with ALL-responsive genes, a Kyoto Encyclopedia of Genes and Genomes (KEGG) pathway mapping tool was used. The KEGG mapper analysis revealed the decreased expression of inflammation-related genes, associated with the Toll-like receptor (TLR) signaling pathway, the phosphoinositol 3-kinase (PI3K)-protein kinase B (AKT) signaling pathway, the nuclear factor (NF)-kappa B signaling pathway, cytokine-cytokine receptor interactions, and the chemokine signaling pathway in the ALL group (Figure 4A,B), indicating that ALL has anti-inflammatory properties. In support of these results, plasma inflammatory cytokine levels decreased in the ALL group (Figure 4C).

### 3.5. Allulose Influenced the Microbiome Composition in DIO Mouse Feces

**Figure 5:**
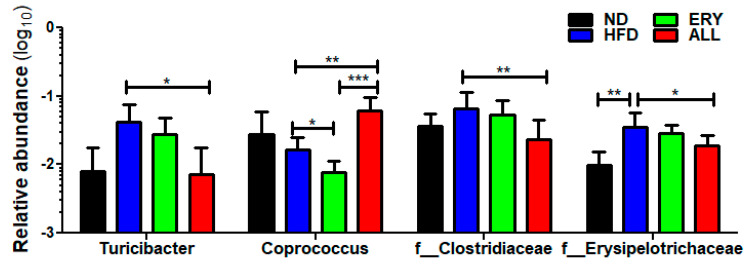
Effects of 16-week D-allulose supplement on microbiota modulation. Relative abundance of *Turicibacter*, *Coprococcus*, *Clostridiaceae* and *erysipelotrichaceae*; Kruskal-Wallis test; * *p* < 0.05, ** *p* < 0.01, *** *p* < 0.001 versus control; ND, normal diet; HFD, high-fat diet (20% fat, 1% cholesterol); ERY (HFD + 5% Erythritol); ALL, (HFD + 5% D-allulose).

The microbiome in feces taxonomy result is suggested in Figure 5. At the genus level, *Turicibacter* population was significantly decreased and *Coprococcus* population was significantly elevated in the ALL group compared with HFD group. Additionally, at the family level, *Clostridiaceae* and *Erysipelotrichaceae* populations were significantly diminished in the ALL group compared with the HFD group. The significantly altered bacteria composition observed in the ALL group compared with the HFD group was examined using Spearman’s correlation coefficient analysis in comparison with the changes in FBG levels. FBG had a significant correlation with the *Turicibacter* genus and *Clostridiaceae* family population (Figure S1). Changes in *Coprococcus* genus and *Erysipelotrichaceae* family did not show any significant correlations with FBG.

## 4. Discussion

In this study, we investigated the anti-diabetic properties of allulose and the potential mechanisms underlying the metabolic regulation of an obesogenic diet, using mRNA-seq data and microbial community results.

Under conditions of excess energy, adipose tissue and adipocytes become expanded for energy storage, which we refer to as fat accumulation [31,32]. Fat accumulation, a hallmark of obesity, not only increases body weight but also induces metabolic dysregulations, such as insulin resistance, dyslipidemia, and hepatic steatosis [33]. Previous studies have suggested that allulose is significantly effective for the amelioration of HFD-induced obesity and its complications by regulating lipid metabolism [22]. In accordance with these results, allulose reduced the plasma lipid profiles and body and adipocyte weights in this study.

Hepatic steatosis has been very closely associated with the long-term consumption of HFD [34]. In this study, the HFD group showed increased liver weights and increased levels of hepatic lipids, compared with the ND group, whereas the allulose group showed reduced liver weight and reduced levels of hepatic lipid contents compared with the other HFD-fed mice. Furthermore, the H&E staining of liver sections indicated that hepatic lipid accumulation was less pronounced in the ALL group compared to HFD group. We observed that the HFD group had significantly increased hepatic lipid levels, as well as hepatic FAS, HMG-CoA reductase, and ACAT activities, compared with those in the ND group. In contrast, the ALL group displayed significantly reduced hepatic FAS, β-oxidation, HMG-CoA reductase, and ACAT activities compared with the HFD group. ALL appears to exert beneficial effects on the initiation or progression of hepatic steatosis. The well-marked improvements in hepatic steatosis, coupled with the decreased adiposity observed in the ALL group, was related with the decreases in the plasma glucose and insulin levels, which was a reflection of ameliorated hepatic insulin sensitivity [35], as evidenced by a reduced area under the curve for the IPGTT. Allulose–induced decreases in hepatic lipid contents appeared to improve hepatic insulin sensitivity. Insulin binds to receptors on hepatocytes, resulting in the inhibition of enzymes involved in gluconeogenesis [36]. Thus, the depressed gluconeogenesis, associated with the decreased levels of hepatic G6Pase and PEPCK activities, was shown to be related with the ameliorated hepatic insulin sensitivity observed in allulose substituted with DIO mice.

The interaction among obesity, insulin resistance, and T2DM has been recognized for decades, and recent extensive research has greatly increased our understanding of the interrelation among these pathophysiologic states [37,38,39]. Low-grade inflammation is an important pathophysiological factor that results in the progression of T2DM, associated with hyperglycemia and insulin resistance [8,40]. Several studies have observed TLR activation, regarded as an alternative activator of obesity mediated inflammation, which has been shown to occur in obesity, mediated through the activation of TLRs in several tissues, and such signaling might contribute to the development of obesity-associated insulin resistance [41,42]. In our study, transcriptome profiling revealed that allulose treatment downregulated the expression of hepatic genes involved in inflammation compared with their expression in the HFD group. Allulose significantly decreased the mRNA expression levels of TLRs, NF-kappa B, PI3K-AKT, cytokines, and chemokines (Figure 4A,B), in addition to plasma inflammatory cytokine levels (Figure 4C). Consistent with these results, our previous study showed that mRNA expression associated with inflammation significantly decreased in the ALL group compared with that in the HFD group, in both hepatic and adipose tissue [43].

The reduction of gut microbial diversity can lead to an increase in the population of pathogenic bacteria, gut inflammation, and the progression of diabetic conditions [44]. The gut inflammatory responses include innate immune response mechanisms, utilizing TLRs, and the production of pro-inflammatory cytokines [45]. In our past study, allulose induced an increase in the alpha and beta diversity of the gut microbiota and the feces absolute total short chain fatty acids amount, which are gut microbial metabolite [46]. In addition, we found significant increases in the relative abundance of *Coprococcus*, whereas the relative abundance of *Turicibacter Clostridiaceae* and *erysilotrichaceae* was markedly diminished in the ALL group compared with the HFD group. Additionally, we found a significant positive correlation between FBG and the abundance of *Turicibacter* and between the abundance of *Clostridiaceae* and FBG. Some studies have demonstrated that *Turicibacter* and *Clostridiaceae* might be important for the abnormal metabolism observed in T2DM. For example, in a comparative study of the intestinal microbial community in T2DM and non-T2DM mice, *Turicibacter* was found to be more abundant in the T2DM mouse model compared with the non-T2DM mouse model [47]. In another study, *Turicibacter* increased following treatment with GLP-1 receptor agonist and DPP-4 inhibitors, which are anti-diabetic drugs [48]. *Clostridiaceae* was enriched in T2DM patients compared with participants without T2DM [49]. Also, when compared with participants with T2DM taking metformin and not taking metformin, the most frequent medication used to treat T2DM, the relative abundance of *Clostridiaceae* was decreased in participants with T2DM taking metformin. 

## 5. Conclusions

In conclusion, the current study demonstrated that allulose supplementation showed a significant effect on mRNA expression and protected the host against HFD-induced obesity and T2DM. These pathologies may be mediated by the regulation of mRNA expression and the alteration of the microbial community. Our findings suggested that allulose ameliorates insulin resistance induced by DIO by modulating the mRNA expression levels in liver tissue and by modulating the gut microbiome.

## Figures and Tables

**Table 1:** Effects of D-allulose supplements for 16 weeks on body weight, body weight gain, food and energy intake, and food efficiency ratio, in C57BL/6J mice fed a high-fat diet.

	ND	HFD	ERY	ALL
Initial BW (g)	19.96 ± 1.13	19.94 ± 1.11	19.61 ± 1.12	19.66 ± 1.41
Final BW (g)	33.34 ± 1.49	41.85 ± 3.19 ***	37.72 ± 3.98	33.11 ± 2.13 ###
Total BWG (g/16 wk)	13.38 ± 1.81	21.91 ± 2.99 ***	18.11 ± 3.50	13.45 ± 2.64 ###
Food Intake (g/day)	3.65 ± 0.10	3.06 ± 0.15	2.62 ± 0.12 $	3.20 ± 0.16
Energy Intake (kcal/day)	14.24 ± 0.39	14.03 ± 0.68	11.48 ± 0.53 $	14.03 ± 0.73
FER	0.03 ± 0.01	0.06 ± 0.00 ***	0.06 ± 0.00	0.04 ± 0.00 ##, @@

Data are presented as the mean ± SD; Kruskal-Wallis test; *** *p* < 0.001, HFD versus ND; ## *p* < 0.01, ^###^ *p* < 0.001, ALL versus HFD; ^$^ *p* < 0.05, ERY versus HFD, ^@@^ *p* < 0.01, ALL versus ERY. ND, normal diet; HFD, high-fat diet (20% fat, 1% cholesterol); ERY (HFD + 5% Erythritol); ALL, (HFD + 5% D-allulose); BW, body weight; BWG, body weight gain; FER, food efficiency ratio = body weight gain/ energy intake per day.

## Supplementary Materials

The following are available online at https://www.mdpi.com/2072-6643/12/7/2113/s1, Figure S1: The correlation between Turcibacter, Clostridiaceae and fasting blood glucose, Table S1: Composition of experimental diets (% of diet, w/w), Table S2. Effects of 16-week D-allulose supplementation on plasma adipokine and cytokine concentrations in C57BL/6J mice fed a high-fat diet, Table S3. he numbers of up- or down-regulated genes in epididymal white adipose tissue D-allulose group compared to the high-fat diet group, Table S4. The list of up- or down-regulated genes in hepatic tissue by allulose among the high-fat diet-responsive genes.
