# Supplementary material for: Anti-Diabetic Effects of Allulose in Diet-Induced Obese Mice via Regulation of mRNA Expression and Alteration of the Microbiome Composition
Source: Nutrients. 2020 Jul 16;12(7):2113. doi: 10.3390/nu12072113 (PMC7400868; doi:10.3390/nu12072113)
Supplement: Supplementary file 1 [file nutrients-12-02113-s001.pdf]

Supplementary Table S1. Composition of experimental diets (% of diet, w/w).

Ingredients (g)	ND	HFD	ALL
Casein	200.00	200.00	200.00
D,L-methionine	3.00	3.00	3.00
Corn starch	150.00	111.00	111.00
Sucrose	500.00	370.00	320.00
Cellulose powder	50.00	50.00	50.00
Corn oil	50.00	30.00	30.00
Lard	-	170.00	170.00
Mineral Mixture (AIN-76) *	35.00	42.00	42.00
Vitamin mix (AIN-76) †	10.00	12.00	12.00
Choline bitartrate	2.00	2.00	2.00
Cholesterol	-	10.00	10.00
tert-Butylhydroquinone	0.01	0.04	0.04
D-allulose	-	-	50.00
Total (g)	1000.0	1000.0	1000.0
Calories (kcal/kg)	3902	4584	4384
Calories (kcal/g)	3.902	4.584	4.384

ND, normal diet; **HFD**, high-fat diet; **ALL**, D-allulose, HFD supplemented with 5% D-allulose. * Mineral mixture (AIN-76) calcium phosphate 500 g/kg, NaCl 74 g/kg, potassium citrate 2220 g/kg, potassium sulfate 52 g/kg, magnesium oxide 24 g/kg, manganous carbonate 3·5 g/kg, ferric citrate 6 g/kg, zinc carbonate 1·6 g/kg, cupric carbonate 0·3 g/kg, potassium iodate 0·01 g/kg, sodium selenite 0·01 g/kg, chromium potassium sulfate 0·55 g/kg, sucrose 118·03 g/kg. † Vitamin mixture AIN-76: thiamin HCl 0·6 g/kg, riboflavin 0·6 g/kg, pyridoxine HCl 0·7 g/kg, niacin 3 g/kg, calcium pantothenate 1·6 g/kg, folic acid 0·2 g/kg, biotin 0·02 g/kg, vitamin B_{12} 1 g/kg, vitamin A (500 000 IU/g) 0·8 g/kg, vitamin D3 (400 000 IU/g) 0·25 g/kg, vitamin E acetate (500 IU/g) 10 g/kg, menadione sodium bisulfite 0·08 g/kg, sucrose 981·15 g/kg.

Supplementary Table S2. Effects of 16-week D-allulose supplementation on plasma adipokine and cytokine concentrations in C57BL/6J mice fed a high-fat diet.

	ND			HFD			ERY			ALL		
Leptin (ng/ml)	4.34	◎◎	2.39	19.58	◎◎	0.20^{**a}	12.89	◎◎	0.30^{a}	12.24	◎◎	0.47^{b}
Resistin (ng/ml)	1.74	◎◎	6.20	2.23	◎◎	0.57^{*a}	1.88	◎◎	0.39^{ab}	1.72	◎◎	1.29^{b}
Adiponectin (ng/ml)	5.27	◎	11.48	4.75	◎◎	0.86^{*b}	4.53	◎◎	0.36^{b}	5.23	◎◎	2.73^{a}
Leptin: Adiponectin	0.83	◎◎	3.93	4.11	◎◎	0.39^{*a}	2.85	◎◎	0.43^{ab}	2.37	◎◎	0.86^{b}
Interleukin 1-β (pg/ml)	75.44	◎◎	10.35	186.03	◎	23.19^{**a}	190.26	◎	47.99^{a}	79.99	±	14.20^{b}
Interleukin 6 (pg/ml)	2.55	◎◎	0.22	4.48	◎	0.26^{**a}	4.65	◎	0.43^{a}	3.50	◎	0.16^{b}
Interleukin 10 (pg/ml)	34.42	◎◎	6.68	38.49	◎	4.40	36.35	◎	8.42	39.72	◎	6.49
Interferon-γ (pg/ml)	7.30	◎◎	0.75	12.08	◎	1.88^{**a}	11.76	◎	1.94^{a}	7.87	◎	1.15^{b}
Monocyte chemoattractant protein 1 (pg/ml)	503.03	◎◎	10.25	553.60	◎	13.37^{**a}	533.57	◎	12.89^{ab}	450.51	◎	9.39^{b}
Tumor necrosis factor–α (ng/ml)	335.13	◎◎	0.05	426.60	◎	0.14^{**a}	401.00	◎	0.06^{a}	329.52	◎	0.06^{b}

Data are presented as the mean ± SE. Significant differences between HFD versus ND are indicated; **$p < 0.01$. ab Means not sharing common letters in the same row are significantly different among the high-fat diet groups, at $p < 0.05$. ND, normal diet (AIN-76); HFD, high-fat diet (AIN-76, 20 % fat, 1% cholesterol); ERY (HFD + 5% Erythritol); ALL, (HFD + 5% D-allulose).

Supplementary Table S3. The numbers of up- or down-regulated genes in epididymal white adipose tissue D-allulose group compared to the high-fat diet group.

Number of differentially expressed genes identified by mRNA-seq analysis						
**Cut off**	**HFD vs ND**		**ALL vs ND**		**ALL vs HFD**	
**FDR < 0.05 & fold change ≥ 2**	**up**	**1285**	**up**	**201**	**up**	**178**
	**down**	**370**	**down**	**292**	**down**	**812**
	**total**	**1655**	**total**	**493**	**total**	**990**
	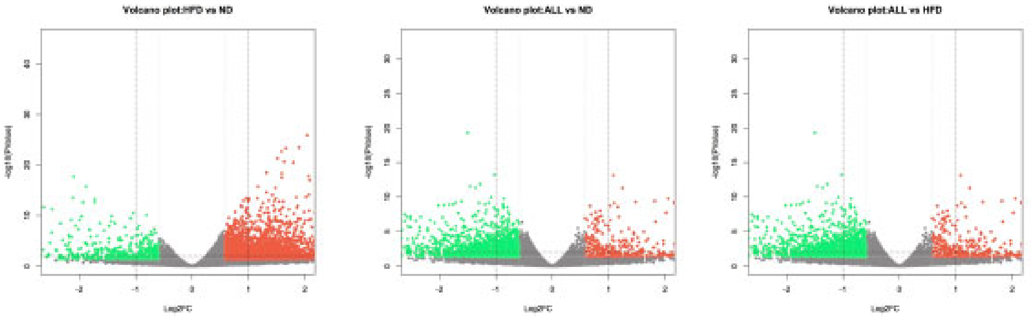					

Differentially expressed genes based on HFD vs ND, ALL vs HFD comparison among to FDR <0.05, fold change ≥2. ND, normal diet (AIN-76); HFD, high fat diet (AIN-76, 20% fat, 1% cholesterol); ALL, (HFD+5% D-allulose).

Supplementary Table S4. The list of up- or down-regulated genes in hepatic tissue by allulose among the high-fat diet-responsive genes.

**Numbers of differentially expressed genes identified by mRNA-seq analysis**	
**Among the 1099 genes up-regulated by HFD**	
**Up 1**	Obp2a
**Down 175**	Aatk, Abcc12, Acod1, Acyp2, Adgrg2, Adgrv1, Anxa2, Apol10b, Aqp7, Atp6v0d2, Atp8b5, B430306N03Rik, Batf3, BC023105, Cbr3, Ccl19ps3, Ccl7, Ccr2, Cd207, Cd274, Cd300lb, Cd52, Ces1g, Chil3, Chst11, Cidea, Ciita, Clec12a, Clec4e, Clec4n, Clec7a, Col12a1, Col3a1, Cotl1, Cpxm1, Csf2rb2, Cxcl10, Cxcl14, Cxcl9, Cyp2a22, Cyp2b13, Cyp2c55, Cyth4, Dcst2, Dfna5, Dgkg, Dhrs9, Diaph3, Dock2, Ear2, Eda2r, Eif4e3, Ephb2, Esco2, Evi2a, Fabp3, Fabp4, Fam65b, Fam83a, Fam83f, Fbxo40, Fgl2, Fgr, Fosb, Gas7, Gbp10, Gbp2, Gbp5, Gbp6, Gins2, Gm10010, Gm12250, Gm13415, Gm15411, Gm16409, Gm37844, Gm44619, Gpr141, H2-DMa,H2-DMb1,H2M2, Haus8, Hk2, Hk3, Ifi209, Ifi211, Ifi27l2b, Ifit2, Igtp, Il27, Insl6, Itga4, Itgb2, Ivl, Kbtbd11, Kif14, Klrk1, Krt8, Ldb3, Lgals1, Lgals3, Limk1, Lrrc14b, Lum, Ly6d, Ly75, Ly86, Ly9, Lyz2, Mas1, Matn2, Mfap3l, Mfi2, Mki67, Mlkl, Mmp12, Mmp13, Mpeg1, Ms4a4c, Ms4a6c, Msrb3, Mthfd1l, Mybl1, Nipa1, Olfr56, Orm2, Orm3, Pcdh18, Plac8, Plek, Plin4, Psd4, Rassf4, S1pr2, Saa1, Saa2, Samsn1, Sdcbp2, Serpina7, Serpinb10, Serpinb9, Sirpb1a, Slamf8, Slc13a3, Slc13a4, Slc15a3, Slc1a4, Slc22a27, Slc28a1, Slc38a1, Slc39a4, Slc41a2, Smpd3, Smpdl3b, Sort1, St18, Tgtp1, Tgtp2, Tifa, Tnc, Tnfrsf19, Top2a, Tpm2, Trem1, Trp53cor1, Ttc39a, Ttc39aos1, Tubb6, Tyrobp, Uap1l1, Ubd, Vldlr, Vsig8, Zfp831, Zfp984
**Among the 158 genes up-regulated by HFD**	
**Up 15**	Cyp4a12b, Cyp4a14, E030018B13Rik, Eif4ebp3, Gm28438, Gm43314, Gm44117, Gm7463, Grm8, Hsd3b5, Lrp2, Moxd1, Mup21, RP23-306P12.3, Trpm5
**Down 4**	Idi1, Pcsk9, RP23-337J18.2, Ugt1a5

Differentially expressed genes based on HFD vs ND, ALL vs HFD comparison among to FDR <0.05, fold change ≥2. ND, normal diet (AIN-76); HFD, high fat diet (AIN-76, 20% fat, 1% cholesterol); ERY (HFD+5% Erythritol); ALL, (HFD+5% D-allulose).

A

Genus level		Turicibacter	Coprococcus
FBG	Spearman Correlation	0.359*	-0.160
	Significance (2-tailed)	0.049	0.366
	N	34	34
**Family level**		**Clostridiaceae**	**Erysipelotrichaceae**
FBG	Spearman Correlation	.360*	-0.107
	0.546	0.036	0.024
	N	34	34

B

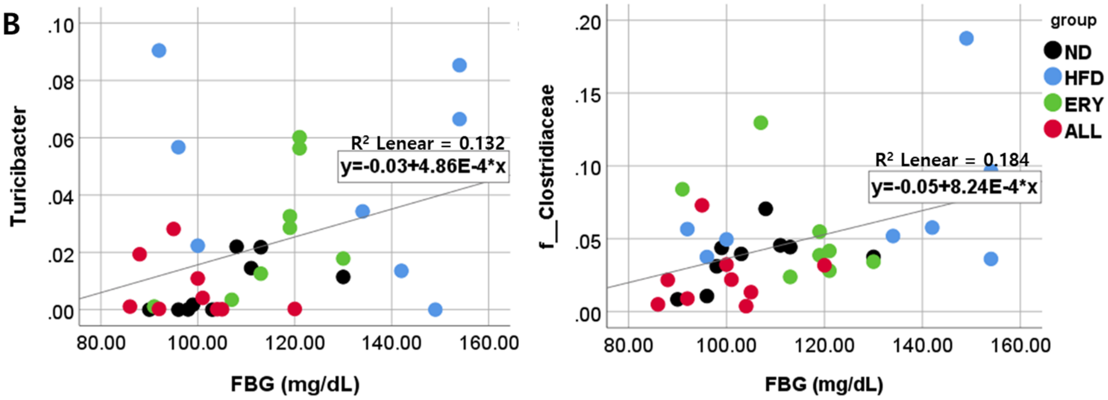

Supplementary Figure S1. The correlation between Turcibacter, Clostridiaceae and fasting blood glucose.
